# Alendronate, a double-edged sword acting in the mevalonate pathway

**DOI:** 10.3892/mmr.2015.3957

**Published:** 2015-06-18

**Authors:** PAOLA MAURA TRICARICO, MARTINA GIRARDELLI, GIULIO KLEINER, ALESSANDRA KNOWLES, ERICA VALENCIC, SERGIO CROVELLA, ANNALISA MARCUZZI

**Affiliations:** 1 Department of Medicine, Surgery and Health Science, University of Trieste, Trieste I-34127, Italy; 2 Department of Advanced Diagnostic and Clinical Trials, Institute for Maternal and Child Health, IRCCS 'Burlo Garofolo', Trieste I-34137, Italy

**Keywords:** alendronate, mevalonate kinase deficiency, programmed cell death, cytokines

## Abstract

Aminobisphosphonate aledronate is a compound commonly used clinically for the treatment of osteoporosis and other bone diseases, as a result of it preventing bone resorption. However, in previous years it has also been used to obtain cellular and animal models of a rare genetic disorder termed Mevalonate Kinase Deficiency (MKD). MKD is caused by mutations affecting the mevalonate kinase enzyme, in the cholesterol pathway and alendronate can be used to biochemically mimic the genetic defect as it inhibits farnesyl pyrophosphate synthase in the same pathway. Despite evidence in favor of the inhibition exerted on the mevalonate pathway, there is at least one clinical case of MKD in which alendronate improved not only skeletal and bone fractures, as expected, but also MKD clinical features. Based on this finding, the present study assessed the anti-inflammatory properties of this aminobisphosphonate *in vitro.* No anti-inflammatory effects of alendronate were observed in the *in vitro* experiments. Since MKD lacks specific treatments, these results may assist scientists and physicians in making the decision as to the most suitable choice of therapeutic compounds for this neglected disease.

## Introduction

Alendronate (Ald), is a molecule belonging to the amino-bisphosphonate family and is commonly used in the clinical treatment of osteoporosis and other bone disorders, including Paget's disease (1,2). Ald acts by reducing the resorptive activity and inducing accelerated programmed cell death of osteoclasts. Besides the well-known antibone resorption activity, the side effects of aminobisphosphonates have been investigated previously and contrasting findings report their pro-inflammatory effects, renal toxicity and adverse reaction in the upper gastrointestinal tract of patients and animal models, although with certain differences depending on the compound and the type of administration (3–6).

These effects are caused, at a molecular level, by the inhibition of the farnesyl pyrophosphate synthase enzyme in the mevalonate pathway. The decreased number of prenylated GTP-binding proteins anchored to the membrane of osteoclasts (7,8) may induce apoptosis and therefore, reduce bone resorption.

**Figure 1:**
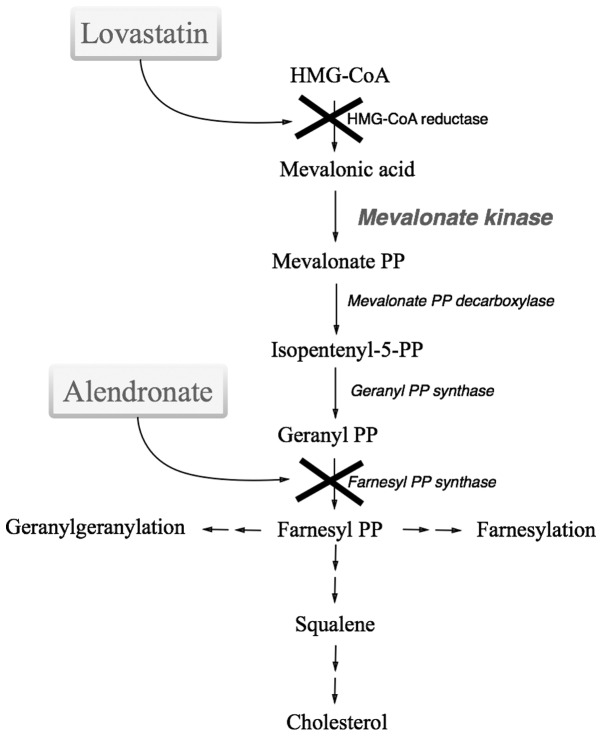
Schematic representation of the mevalonate pathway. The compounds used in the present study are indicated at the side of the pathway. Treatment with alendronate and lovastatin biochemically inhibited the pathway.

Malfunctions in the post-translational modification known as prenylation and the overproduction of several pro-inflammatory cytokines, including IL-1β, are also suggested to be at the basis of Mevalonate Kinase Deficiency (MKD) (9). MKD is an autosomal recessively-inherited disease (OMIM #610377) caused by mutations in the *MVK* gene (12q24.11), which encodes the enzyme, mevalonate kinase (MK) in the mevalonate pathway (Fig. 1) (10–13).

Our previous studies developed cellular and animal models of MKD obtained following administration of the aminobisphosphonate, Ald or lovastatin (Lova). These inhibit the mevalonate pathway and allow partial reproduction of the biochemical defect characterizing patients with MKD (14,15).

However, Cantarini *et al* (16) described a case report in which Ald was administered to a patient with MKD to prevent skeletal and bone fractures and this treatment markedly rescued the inflammatory symptoms and led to a disease remission period of several months (16). The positive effects of Ald suggested that this may be used as a potential therapeutic drug for MKD and not exclusively for bone disorders (16). However, this raises the issue of the apparently opposite roles of Ald in MKD. This aminobisphosphonate appears to exhibit contrasting effects, while having been reported to improve the clinical features of one patient, it has been extensively used to inhibit the mevalonate pathway *in vivo* and *in vitro* (14,15).

The intriguing observation that Ald antagonizes the pro-inflammatory effects of the inhibited mevalonate pathway, prompted the present study to re-assess the activity of this compound, using an MKD cellular model (murine Raw 264.7 monocyte-macrophage cell line) and monocytes isolated from two patients with MKD.

Additionally, to avoid compound-dependent results and dissipate any question or controversy of the findings obtained, the pathway was inhibited using two different compounds, Ald and Lova. Once the pathway was inhibited, the acute phase was mimicked by administering a pro-inflammatory stimulus, lipopolysaccharide (LPS), shortly followed by the therapeutic administration of Ald. The analysis of four pro-inflammatory cytokines, interleukin (IL)-1α, IL-1β, IL-6 and tumor necrosis factor (TNF)-α, and programmed cell death (PCD), was used to assess the potential anti-inflammatory effects of Ald on these cell models of MKD.

## Materials and methods

### Chemicals

Unless otherwise stated, the reagents were purchased from Sigma-Aldrich (Milan, Italy). LPS (*E. coli* serotype 055:B5; 1 mg/ml stock in H_2_O), Ald (30 mM) and Lova (50 mM) were dissolved in saline solution (Diaco SpA, Trieste, Italy).

### Cell culture

The raw 264.7 cells (murine monocyte/macrophage cell line; Sigma-Aldrich) were cultured at 2.5×10^5^ cells/ml in Dulbecco's modified Eagle's medium, supplemented with 10% fetal bovine serum (FBS; Euroclone Spa, Milan, Italy) and 100 *µ*M Ald or 20 *µ*M Lova for 20 h at 37°C in a 5% CO_2_ incubator. Following incubation, 10 *µ*g/ml LPS was added for an additional 24 h. Where appropriate, Ald was added at three different concentrations (25, 50 or 100 *µ*M) together with LPS in order to analyze its potential anti-inflammatory properties.

Two patients with MKD were diagnosed during the first year of life and followed-up at the Institute for Maternal and Child Health, IRCCS 'Burlo Garofolo', Trieste, Italy. The MKD diagnosis was confirmed by genetic analysis (Table I). These two patients were recruited to the present study at the age of 24 and 13. They had no recurrent infections and were not in the acute phase of the disease at the time of enrollment. The present study was approved by the ethical and scientific review board of the Institute for Maternal and Child Health, IRCCS 'Burlo Garofolo' (no. 185/8; 19/08/2008). For a child to be eligible, informed consent had to be obtained from the parents or caregivers. Monocytes were isolated from the two patients with MKD by selection with monoclonal CD14 antibody (mouse IgG2a)-conjugated microbeads (Miltenyi Biotec, Bergisch Gladbach, Germany), performed with manual columns, according to the manufacturer's instructions. The cells were subsequently cultured at 2.5×10^5^ cells/ml in RPMI-1640 medium, containing 10% FBS (Euroclone Spa) and 1 *µ*g/ml LPS for 24 h. As for the Raw 264.7 cells, 50 *µ*M Ald was added together with LPS, where appropriate

At the end of the incubation periods, the supernatant was collected for the cytokine assay and the cells were pelleted for the PCD assay.

### PCD assay

The PCD of the Raw 264.7 cells and the patient isolated monocytes were monitored by flow cytometry using double staining with Annexin V-fluorescein isothiocyanate and propidium iodide (PI; Apoptosis Detection kit; Immunostep, Salamanca, Spain), according to the manufacturer's instructions. The fluorescence was measured with a Cyan ADP cytometer and Summit version 4.3 software (Beckman Coulter, Fort Collins, CO, USA), and was subsequently analyzed with FlowJo 7.6 software (TreeStar Inc., Ashland, OR, USA). This technique was used to assess the effect of the treatments on cell viability. Debris were excluded from the plot based on the scatter (FSC vs. SSC) and the apoptotic (Annexin V positive, A^+^; PI negative, PI^−^ and positive, PI^+^) and the necrotic (A^−^ and PI^+^) cells were characterized based on the fluorescence emitted.

### Cytokine production assay

The analysis of four pro-inflammatory cytokines, IL-1α, IL-1β, IL-6 and TNF-α, was performed on culture medium from the Raw 264.7 cells and the patient isolated monocytes, using magnetic bead-based multiplex immunoassays (Bio-Plex; Bio-Rad Laboratories, Milano, Italy), according to the manufacturer's instructions. The data from the reactions were acquired using the Bio-Plex 200 reader, a digital processor managed the data output and Bio-Plex Manager 6.0 software (Bio-Rad) presented data as the Median Fluorescence Intensity and concentration (pg/ml).

### Statistical analysis

The statistical significance was calculated using one-way analysis of variance and Bonferroni post-hoc test correction in the case of multiple comparisons, using GraphPad Prism v5.0 software (GraphPad Software Inc., La Jolla, CA). The data are expressed as the mean ± standard deviation. P<0.05 was considered to indicate a statistically significant difference.

## Results

### PCD

**Figure 2:**
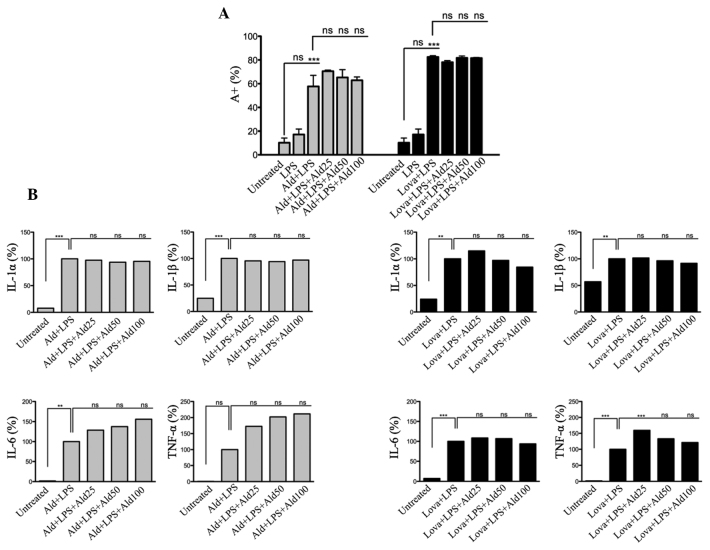
Raw 264.7 cells were incubated with 100 *µ*M Ald (left, grey) or 20 *µ*M Lova (right, black) and subsequently with 10 *µ*g/ml LPS and Ald (25, 50 or 100 *µ*M). (A) The percentage of apoptotic cells was detected by positive Annexin V staining (A^+^) and the data are expressed as the mean ± standard deviation (B) The levels of IL-1α, IL-1β, IL-6 and TNF-α in the supernatant was assessed. The data are demonstrated as the percentage of levels compared to the standard value (100%). One-way analysis of variance and Bonferroni post-hoc test was performed for three independent experiments (^*^P<0.05, ^**^P<0.01 and ^***^P<0.001, compared with the untreated group). Ald, alendronate; Lova, lovastatin; LPS, lipopolysaccharide; ns, not significant.

The Raw 264.7 cells demonstrated a statistically significant increase in PCD following the addition of a specific inhibitor (Ald or Lova) in addition to LPS. The Ald + LPS-treated or Lova + LPS-treated cells produced comparable results demonstrating that PCD was independent of the compound used to inhibit the pathway (Fig. 2A). Additionally, the production of the pro-inflammatory cytokines, IL-1α, IL-1β, IL-6 and TNF-α, was not normalized by the presence of Ald, regardless of which inhibitor was previously used (Fig. 2B).

### Anti-inflammatory effect of Ald

In order to establish the potential anti-inflammatory effect of Ald, the drug was assessed at different concentrations (25, 50 and 100 *µ*M) in the murine cellular model. No affect was observed at any concentration with regards to the decrease of PCD or the secretion of pro-inflammatory cytokines (Fig. 2A and B).

### Therapeutic activity of Ald

**Figure 3:**
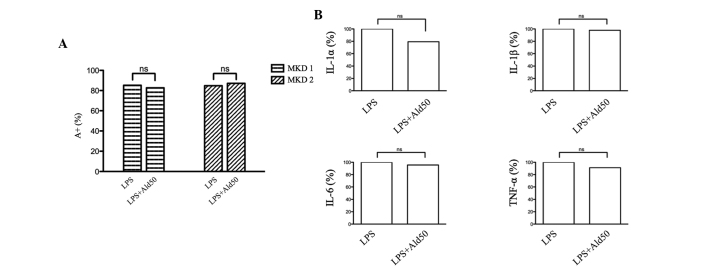
Monocytes from patients with Mevalonate Kinase Deficiency were incubated with 1 *µ*g/ml LPS and 50 *µ*M Ald. (A) The percentage of apoptotic cells were determined by positive Annexin V (A^+^) staining. The data are expressed as the mean. (B) The levels of IL-1α, IL-1β, IL-6 and TNF-α in the supernatant were assessed. The data are demonstrated as the percentage of levels compared with LPS, considered as 100% of cytokine production in the respective experimental setting. One-way analysis of variance and Bonferroni post hoc test was performed for three independent experiments. LPS, lipopolysaccharide; Ald, alendronate; ns, non significant.

The effects of Ald on monocytes isolated from two different patients with MKD (Table I) were assessed. Following the addition of the pro-inflammatory stimulus, LPS, the potential therapeutic activity of Ald was assessed. As previously observed in the murine cell line, Ald revealed no improvement in the PCD in the monocytes from the patients with MKD, however, increased the percentage of apoptotic cells (Fig. 3A).

### Cytokine levels

The production levels of the cytokines IL-1α, IL-1β, IL-6 and TNF-α were determined in the supernatant of the monocytes from the patients. The sensitivity of the specific human kit and their ranges were as follows: IL-1α (1.4-22569 pg/ml), IL-1β (3.2-3261 pg/ml), IL-6 (2.3-28880 pg/ml) and TNF-α (5.8-95484 pg/ml). The results were in the range of the limits of quantification and therefore, the data obtained demonstrated the inefficacy of Ald treatments in decreasing inflammation (P>0.05) (Fig. 3B).

## Discussion

Despite the positive result described by Cantarini *et al* (16) in their case study, the present study failed to reproduce the anti-inflammatory effects of Ald *in vitro*, using a murine cell line or monocytes isolated from two patients with MKD. By contrast, treatment with Ald continued to be associated with increased levels of PCD and the production of inflammatory cytokines, suggesting the lack of anti-inflammatory activity for this compound, at least *in vitro*. A possible explanation for these contrasting findings may reside in the different backgrounds of the MVK gene. Indeed, the patient described by Cantarini *et al* and the two patients in the present study carry different mutations, the first being homozygous for V377I (16) and the latter being compound heterozygous (S135L/V377I and I268T/V377I).

In addition, patients with MKD exhibit a heterogeneous clinical phenotype, characterized by recurrent episodes of fever, irritability, lymphadenopathy, abdominal pain, diarrhoea and skin rash, which differs in terms of intensity and frequency from one patient to the other. Additionally, patients with MKD also exhibit marked variability in the response to therapies (statins and biological drugs) designed to rescue the inflammatory phenotype (17–19). According to Hoffmann *et al* (20), it is inappropriate to administer statins and/or aminobisphosphonate to patients with MKD exhibiting a genetically determined inhibited mevalonate pathway. However, the same author, reported discordant effects following the administration of statins. Indeed, certain patients with MKD exhibited an improvement of the clinical features, while other patients exhibited detrimental effects, including a marked increase of febrile attacks (20).

Despite isolated cases in which a variety of compounds have been demonstrated to improve the symptoms exhibited by patients, MKD still lacks standardized and targeted therapies and remains a neglected and disease, without a recommended therapeutic agent. Since our previous study reported an *in vitro* model useful to assess aminobisphosphonates, it was suggested to physicians to perform *in vitro* assays on monocytes isolated from patients with MKD. Therefore, a preliminary evaluation of the proper anti-inflammatory action of treatments may be obtained prior to treating patients themselves.

Being aware of all the limitations represented by cell models, in which the mevalonate pathway has been biochemically inhibited, the present study suggested that the *in vitro* model may contribute to identifying a common therapeutic strategy for patients with MKD.

## Figures and Tables

**Table I:** *MVK* gene mutations.

Patient	Mutation
1	S135L/V377I
2	V377I/I268T
